# Photon counting imaging and centroiding with an electron-bombarded CCD using single molecule localisation software

**DOI:** 10.1016/j.nima.2016.03.024

**Published:** 2016-06-01

**Authors:** Liisa M. Hirvonen, Matthew J. Barber, Klaus Suhling

**Affiliations:** Department of Physics, Kings College London, Strand, London WC2R 2LS, UK

**Keywords:** Electron-bombarded CCD, Single photon counting, Single-molecule localisation

## Abstract

Photon event centroiding in photon counting imaging and single-molecule localisation in super-resolution fluorescence microscopy share many traits. Although photon event centroiding has traditionally been performed with simple single-iteration algorithms, we recently reported that iterative fitting algorithms originally developed for single-molecule localisation fluorescence microscopy work very well when applied to centroiding photon events imaged with an MCP-intensified CMOS camera. Here, we have applied these algorithms for centroiding of photon events from an electron-bombarded CCD (EBCCD). We find that centroiding algorithms based on iterative fitting of the photon events yield excellent results and allow fitting of overlapping photon events, a feature not reported before and an important aspect to facilitate an increased count rate and shorter acquisition times.

## 1 Introduction

The detection of single photons is a technique used in many fields of science and technology, including fluorescence microscopy and spectroscopy, bioluminescence studies, optical tomography, DNA sequencing, lidar, quantum information science and encryption, and optical communications both on earth and in space [1], [2], [3], [4]. Photon counting imaging is a well-established low light level imaging technique where an image is assembled from individually detected photons. In conventional photon counting imaging, photon events on the phosphor screen of a microchannel plate (MCP)-based image intensifier are imaged with a charge-coupled device (CCD) or a complementary metal-oxide-semiconductor (CMOS) camera at high frame rates, and many frames are accumulated to build up an image [5]. Photon counting imaging is also possible with electron-bombarded (EB) sensors, where single photoelectrons liberated from the photocathode are accelerated by a high voltage directly into the CCD or CMOS sensor [6] to produce a photon event [7]. These are smaller and less bright than MCP-intensified photon events, due to a generally lower gain of electron-bombarded sensors, with a narrow, voltage-dependent pulse height distribution [8]. EBCCD or EBCMOS-based photon counting imaging avoids distortion of the image due to the coupling of the intensifier to the camera, and image lag due to the phosphor decay time, and there is no need for spectral matching of the camera sensitivity and the phosphor.

A characteristic feature of the photon counting imaging technique is the possibility of calculating the true position of a photon event that covers several pixels with subpixel accuracy – a process termed centroiding [9], [10], [11]. The original centroiding algorithms, based on a simple center-of-mass calculation [11], were developed for implementation in hardware. With the advent of more powerful computers, it became possible to implement increasingly complex centroiding algorithms based on fitting the photon event in software. However, some of the algorithms employed in photon counting imaging are still simple, one-iteration algorithms [12].

In the past decade, photoswitchable and photoactivatable fluorescent probes [13] have allowed the same centroiding principle to be employed in circumventing the diffraction limit in fluorescence microscopy. Single-molecule localisation fluorescence microscopy techniques are based on the activation of a small subpopulation of the fluorescent proteins or fluorophores used to stain the sample. They are imaged and subsequently deactivated before the process is repeated with a different subset of fluorophores [14], [15], [16]. The centroid positions of the fluorescent probes are calculated in each frame, typically by fitting a three-dimensional Gaussian function to the fluorophore׳s point spread function, and the final image is formed by summing many frames. Single-molecule localisation fluorescence microscopy is now a well-established technique, and much effort has been put into the development and optimisation of many different types of centroiding algorithms, including iterative fitting algorithms [17].

We recently reported that single-molecule localisation algorithms produce excellent results when applied to centroiding single photon events imaged with an MCP-intensified CMOS camera [18]. Here, we extend this work and apply super-resolution software for centroiding photon events detected with an EBCCD camera. Moreover, multi-emitter fitting analysis was used for separating overlapping photon events, an important aspect not reported before, which allows an increased count rate and shorter acquisition times.

## 2 Method

**Fig. 1:**
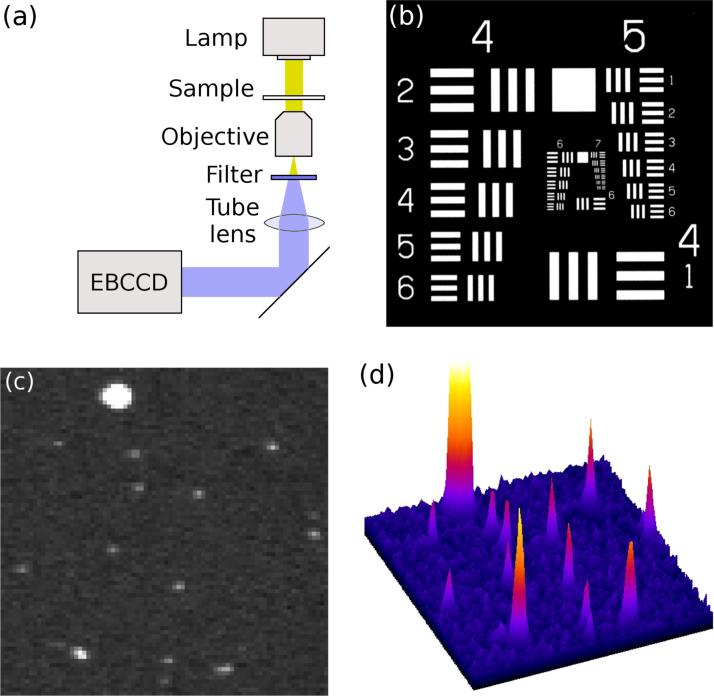
(a) Schematic diagram of the data acquisition setup. (b) Total imaged area of USAF test pattern. (c) An 80×80-pixel area of a raw data frame with single-photon events and an ion event. (d) 3D representation of (c).

Photon counting imaging was performed with a dual mode cooled Hamamatsu C1790-13 EBCCD, with 512×512 pixels and 24×24 μm pixel size. The EBCCD was cooled to −15 °C, and HiPic 7.1.0 software was used for image acquisition with 10 μs exposure time and super-high amplifier gain. The EBCCD was attached to the output port of an inverted Nikon Eclipse TE2000-U microscope, as schematically illustrated in Fig. 1a. For transmission imaging of a 1951 USAF resolution test chart (Fig. 1b), the microscope was used with a 4×0.13 NA air objective (Nikon) and a halogen lamp. For epifluorescence imaging, a cell sample (FluoCells Prepared Slide #1, Molecular Probes) was excited with a pulsed 467 nm diode laser (Hamamatsu PLP-10) and imaged with a 100×1.4 NA oil objective (Nikon). The illumination intensity was adjusted such that single photon events could be observed (Fig. 1c and d).

The frames containing single photon events were processed with ThunderSTORM [19] superresolution imaging plug-in for ImageJ. Due to memory limitations, the USAF test chart data was processed in 6×5000 and the cell data in 3×2000 image stacks. The software first detects the events from the noise background, and an approximate localisation algorithm locates the center pixel of each event. A sub-pixel localisation algorithm then calculates the center of the events with greater resolution.

The software camera parameters were set to 80.0 nm pixel size and 36 photoelectrons per A/D count. The base level varied between image stacks due to fluctuations in the EBCCD temperature, and was set to the average minimum grey value for the image stack in the range of 100–140 A/D counts. A wavelet (b-spline) image filter was applied with order of 3 and scale of 2.0. For the approximate localisation of the events, the centroid of connected components method was used with a peak intensity threshold (PIT) of 2⁎std(Wave.F1) for the USAF test chart data, and a PIT of 1.5⁎std(Wave.F1) for cell data, with the watershed algorithm enabled for all data.

All sub-pixel localisation methods offered by ThunderSTORM (maximum likelihood (ML) and least squares (LS) fitting with both Gaussian (G) and integrated Gaussian (IG) point-spread function (PSF), centroid of local neighbourhood, and radial symmetry) were tested, with fitting parameters optimised for maximum photon count and minimum likelihood of false photon event recognition.

For the best results, ML fitting was used with a Gaussian PSF, with standard deviation (SD) set to 1.0 pixels. For fast processing with adequate results the PSF fitting radius was set to 2 pixels, and for optimal photon detection and separation of overlapping events the radius was set to 7 pixels. Multiple-emitter fitting analysis (MFA) was tested with a maximum of 2 molecules per fitting region with a model selection threshold (*p*-value) of 10^−6^. When MFA was enabled, ThunderSTORM׳s “remove duplicates” post-processing tool was applied with a distance threshold of 160 nm, and “intensity>4000” filter was applied to the USAF data and “intensity>3000” filter to the cell data.

Results are also shown for LS fitting method with an IG PSF with a 3 pixel fitting radius and 1.6 pixel SD, and a radial symmetry localisation method with 2 pixel estimation radius.

## 3 Results and discussion

Typical single photon events detected with the EBCCD are shown in Fig. 1c and d. The central peak is high with small wings: during the diffusion of the electrons from the back of the sensor to the front, the charge spills over into adjacent pixels. Brighter, larger ion events are also detected, caused by a photoelectron ionising a residual gas molecule in the imperfect vacuum inside the EBCCD tube, leading to the resulting ion being accelerated towards the photocathode (Fig. 1c and d, top). These ion events cause problems with event recognition algorithms that find a threshold for each frame separately: the high brightness causes the threshold to be set too high, and the photon events are discarded as noise. The raw data was therefore preprocessed using ImageJ׳s tools by setting the intensity of all bright pixels in the ion events to a grey value slightly above the maximum intensity of the photons events. The ion events are then incorrectly localised as photon events, but due to the relatively rare occurrence of ion events compared to photon events (i.e. an ion event every few frames) this does not have a noticeable effect on the results.

**Fig. 2:**
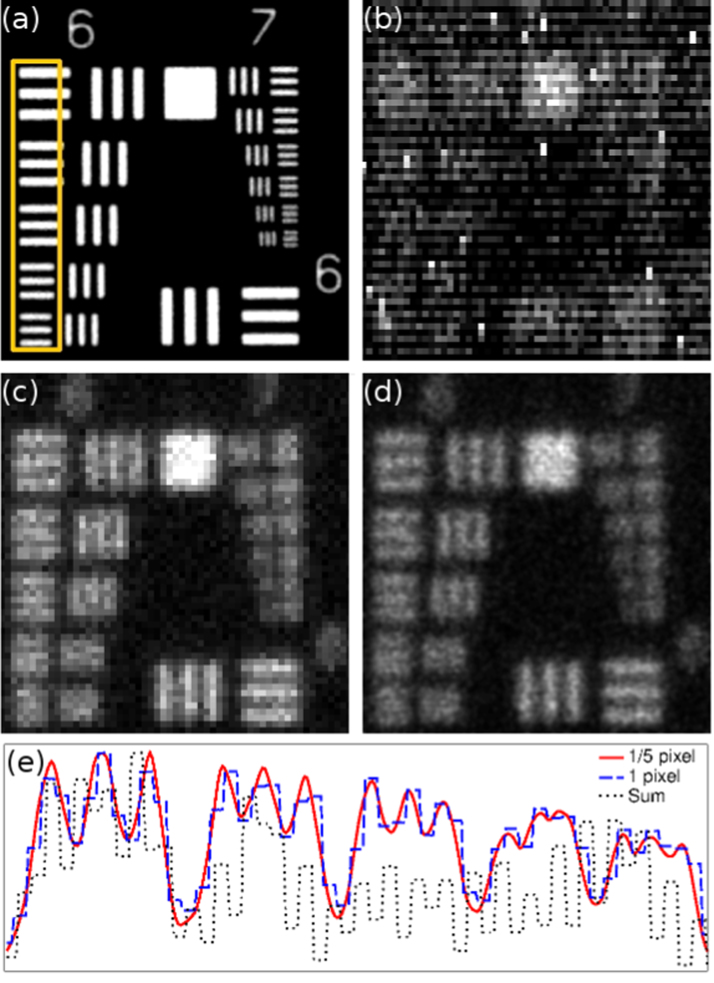
Images of USAF test pattern obtained by photon counting imaging with an EBCCD. (a) Zoomed area of USAF test pattern, (b) sum of the 30,000 frames, (c) 1-pixel centroiding, (d) 1/5-pixel centroiding using ML with Gaussian PSF, and (e) line profiles of the area indicated in (a) by the orange rectangle. (For interpretation of the references to colour in this figure caption, the reader is referred to the web version of this paper.)

Photon counting images of the USAF test chart with 30,000 frames are shown in Fig. 2. The sum of the frames without any processing includes the camera noise and produces a very noisy image (Fig. 2b), while centroiding with one pixel accuracy, where the photon is assigned to the center pixel of the event and the edges ignored, removes the camera background and produces a much clearer image (Fig. 2c). Each pixel was then divided into 5×5 subpixels, and each photon event was assigned a sub-pixel according to the centroid position calculated by sub-pixel localisation algorithm. This method of centroiding with 1/5-pixel accuracy results in an even better image (Fig. 2d) as it seems to recover some of the resolution lost by the electron diffusion in the sensor, as shown in Fig. 2e.

The mismatch between the photon event shape and the centroiding function can lead to fixed pattern noise (FPN) which can be seen as bright and dark stripes in the centroided image [10]. The level of FPN can be quantified by

(1) $$\mathrm{FPN}=\frac{N_{\max}-N_{\min}}{N_{\mathrm{mean}}}\times 100\%$$

where *N*_*max*_, *N*_*min*_, and *N*_*mean*_ are the maximum, minimum and average number of counts in the 5×5 array of subpixel positions, respectively. For minimum FPN, this number should be as low as possible. The EBCCD photon events are asymmetric due to the CCD read-out [8]. Unlike the photon events on an image intensifier screen which can imaged at high magnification for detailed analysis [20], the EBCCD events only cover very few pixels, and a more detailed analysis of the EBCCD photon event shape was therefore not undertaken.

**Fig. 3:**
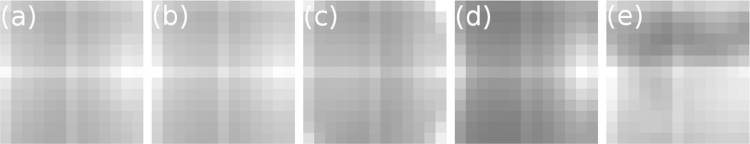
Distributions of centroided photon positions within a pixel for the USAF test pattern data set. (a) ML with Gaussian PSF and 2-pixel fitting radius, and 7-pixel fitting radius (b) without and (c) with MFA; (d) weighted LS with Integrated Gaussian PSF; and (e) radial symmetry.

Several of ThunderSTORM׳s centroiding algorithms were tested to find an algorithm that leads to a minimum amount of FPN (see Table 1). The distributions of centroid positions for the USAF test chart data set are shown in Fig. 3. They are obtained by overlaying the centroid positions of all pixels, divided into a 13×13 grid. Maximum likelihood (ML) fitting with a Gaussian PSF produces the most uniform distribution of localised positions (Fig. 3a–c), as well as the finding the highest number of photons (see Table 1). As reported previously [8], the horizontal widening of the photon events, most likely caused by the CCD read-out, causes a bias in the centroided positions and photon events are more likely to be found towards the right edge of the pixel. Other methods produce results with a similar distribution of centroid positions but with higher FPN and lower photon count (an example of a weighted LS fit with an integrated Gaussian PSF is shown in Fig. 3d), with the exception of the radial symmetry method, which changes the bias to the vertical direction (Fig. 3e).

**Fig. 4:**
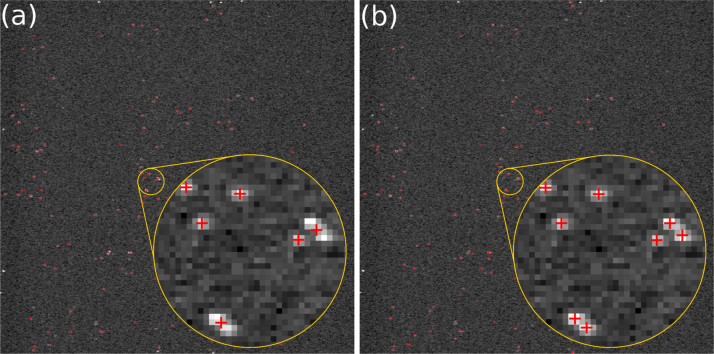
A raw frame of USAF test pattern data with localised photon positions marked with red crosses. Overlapping events that are counted as one event without multiple-emitter fitting analysis (MFA) (a) are resolved with MFA enabled (b). (For interpretation of the references to colour in this figure caption, the reader is referred to the web version of this paper.)

ThunderSTORM׳s MFA option produces excellent results with recognising and separating overlapping EBCCD photon events, as shown in Fig. 4. For the USAF test pattern data containing an average of 150 photons/frame, the photon count increases 2% with MFA enabled.

**Fig. 5:**
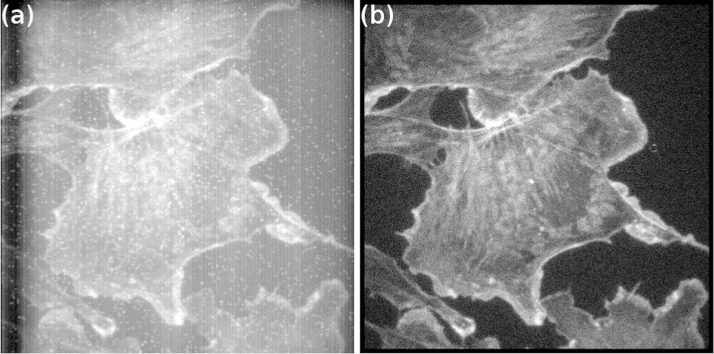
Images of Alexa-488-stained F-actin in a BPAE cell obtained by photon counting imaging with an EBCCD. (a) Sum of 6000 frames and (b) centroided image. Centroiding removes the camera background and artefacts caused by ion events, and recovers resolution lost during electron diffusion process in the sensor, producing a clearer image.

Centroiding was then applied to higher photon density fluorescence microscopy data of F-actin in a fixed BPAE cell labelled with Alexa-488 (Fig. 5). The 6000-frame data set contains an average of 460 photons/frame. With 7-pixel PSF fitting radius and MFA enabled, the photon count increases 13.0% compared to using 2-pixel fitting radius, and 5.2% compared to 7-pixel fitting radius without MFA. Fitting of multiple PSFs is time-consuming and the processing time increases significantly with MFA enabled, as expected (see Table 1). However, for biological imaging the image acquisition time is a critical parameter, and the separation of overlapping events can potentially lead to significant reduction in image acquisition times.

## 4 Conclusion

With the recent developments in single-molecule localisation microscopy, there is a large choice of well-characterised and user-friendly software packages and algorithms freely available [17]. We have shown previously that when applied to centroiding photon counting imaging data from an MCP-intensified camera system, these programs can produce results with better resolution, lower FPN, and in some cases even better event recognition than the traditional single-iteration photon centroiding algorithms. In this work we have shown that these programs produce excellent results when applied to centroiding smaller and less bright photon events from an EBCCD camera with significant background noise. Moreover, we have also demonstrated that multi-emitter fitting analysis can separate overlapping photon events – an important consideration for photon counting imaging fluorescence microscopy where the count rate is usually limited by the need to avoid overlapping photon events.

## Figures and Tables

**Table 1:** Processing time, number of localised photons, and fixed pattern noise for different centroiding methods. Radius=PSF fitting radius in pixels.

Sample	Method	Radius	Frames	Time (min)	Photons	FPN (%)
USAF	LS (IG)	3	30,000	67	4,446,206	154
USAF	RS	2	30,000	53	4,446,206	91
USAF	ML (G)	2	30,000	106	4,452,295	70
USAF	ML (G)	7	30,000	221	4,460,470	64
USAF	ML (G) MFA	7	30,000	3875	4,542,958	59

Cells	ML (G)	2	6000	21	2,654,522	71
Cells	ML (G)	7	6000	93	2,852,941	67
Cells	ML (G) MFA	7	6000	1716	2,999,894	64
